# “Inert” and Active Ingredients: Séralini Responds

**Published:** 2005-10

**Authors:** Gilles-Eric Séralini

**Affiliations:** Laboratoire de Biochimie et Biologie Moleculaire Université de Caen, Caen, France, E-mail: criigen@ibfa.unicaen.fr

Surgan raises interesting points in his analysis. This interest has been confirmed by reactions of agriculture authorities all over the world after publication of the article by Richard et al. (2005).

Indeed, scientific problems do exist in the registration of pesticides today, when chronic toxicity tests are conducted with the active ingredient alone—which is generally the case. First of all, chemists from companies may work hard for several years to find the right formulation that best amplifies the effects of the active ingredient. his formulation will allow penetration and stability and/or bioaccumulation of the active ingredient within plant, fungi, or insect cells, for instance, to reach the best toxicity. If there are any side effects in other animal or human cells, these will be also amplified by adjuvants, and thus not measured in chronic toxicity tests with the active ingredient alone. The active compound absorption by skin is generally calculated in the presence of formulated adjuvants, but this is clearly a short-term study and not sufficient to detect, for example, endocrine disruption or carcinogenesis, possibly promoted *in vivo* by the described synergy. This should even necessitate further care in case of the use of formulated products such as glyphosate-based herbicides on tolerant, edible plants.

As a matter of fact, most genetically modified crops have been modified and selected only to tolerate high-formulated herbicide absorption, but the plants are not submitted for registration requiring chronic toxicity studies involving long-term feeding of animals. Moreover, in the case of environmental pollution, active pesticide ingredients may encounter detergents or other lipohilic xenobiotics with comparable effects other than those of their own adjuvants, for instance, forming microvesicles to penetrate the cells. These combined effects should also be taken into account in authorized thresholds of pollution in order to avoid effects on wildlife or humans.
